# Methods for single-pair *Ascaridia galli* genetic crosses

**DOI:** 10.17912/micropub.biology.002043

**Published:** 2026-03-17

**Authors:** JB Collins, Erik C Andersen

**Affiliations:** 1 Department of Biology, Johns Hopkins University, Baltimore, MD, USA

## Abstract

Ascarid parasites infect a wide range of hosts, causing significant clinical and economic impacts. However, genetic tools for studying ascarid biology remain limited. We optimized genetic crosses using *Ascaridia galli* , a common ascarid of chickens. Sexually immature larval parasites were recovered from donors, transferred to gelatin capsules, and then given orally to recipients. We successfully established single-pair matings in 32% of crossing attempts. This method to control genetic crosses further establishes the avian model for ascarid research and will enable future studies to create a high-quality reference genome, inbreed anthelmintic resistant and sensitive lines, and investigate host-pathogen interactions.

**Figure 1. Overview of crossing methods:**
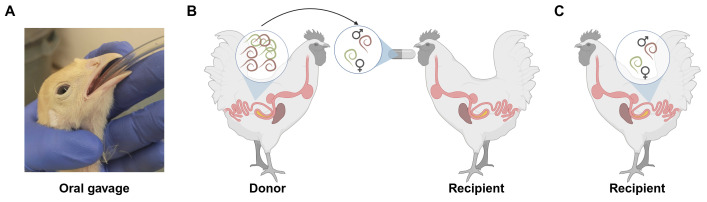
A) Gavage of a chick with *Ascardia galli* embryos. Chicks are held with the head tilted upward, and a gavage tube is guided into the esophagus, where embryos are delivered. B) Larval (L4) parasites are recovered from the small intestine of a donor animal, encapsulated in a gelatin capsule to create a cross, and then the capsule is delivered orally to a recipient animal. C) Three weeks after crossing, the recipient animals are necropsied to determine if the cross was established in the small intestine. Figure made using BioRender.

## Description

Ascarid parasites are the most common nematode parasite of humans, infecting an estimated 1.2 billion people annually, and are nearly ubiquitous in many veterinary species (Centers for Disease Control and Prevention, 2020; C. Holland et al., 2022; Yazwinski et al., 2013). Infections are typically associated with poor growth and development. Heavy worm burdens can cause intestinal rupture and death (Ikeme, 1971). Despite the prevalence and severity of infections, resources for studying ascarid biology are less readily available than for other nematode species (Collins & Andersen, 2022; Wolstenholme et al., 2024). The free-living nematode *Caenorhabditis elegans* has been essential to advance our understanding of closely related parasitic nematode species such as *Haemonchus contortus* , a parasite of small ruminants, which has a high-quality genome that adds further synergy to comparative genomic studies (Britton et al., 2016; Doyle et al., 2020). However, ascarids lack a closely related free-living species, necessitating the development of a model system for ascarid research.&nbsp;

Most ascarid research focuses on *Ascaris suum* , an ascarid of swine (C. V. Holland et al., 2013; Wang, 2021). However, swine are expensive hosts and difficult to study because of their rapid growth. Our work has focused on developing poultry ascarids as a model system. Poultry offer an inexpensive vertebrate host that is easily manipulated, facilitating larger-scale experiments with greater levels of replication. As part of the development of genetic and genomic resources for an ascarid model, we optimized genetic crosses using *Ascaridia galli* , a common ascarid of chickens. Previous methods for the crossing of parasites used in *H. contortus* relied on surgical transplantation between animals, making replication of crosses difficult and costly (Doyle et al., 2019). Here, we used gelatin capsules, enabling crosses without anesthesia or surgery, thereby greatly reducing costs and discomfort.&nbsp;

We conducted two sets of experiments in which week-old chicks were infected with *A. galli* to serve as donors for genetic crosses ( Figure 1A ). Parasites were allowed to develop to the fourth larval stage (L4) (21 days post-infection). L4 parasites were used for crossing, because larvae can be sexed but are not mature enough to have already mated. Donor animals were humanely euthanized, and L4 parasites were recovered from the small intestine of donor animals, sexed, and gelatin capsules for crosses were prepared for transplantation to recipient animals ( Figure 1B ). Capsules were given to recipient animals, where, once dissolved, parasites would make their way to the small intestine and mate to produce cross progeny. Three weeks after delivery of cross animals to recipients, chickens were humanely euthanized, and ascarid parasites were recovered from the small intestine to determine the success of crossing ( Figure 1C ). In the first experiment, crosses were performed with 1:1 male to female sex ratios (10 replicates) and 5:5 sex ratios (9 replicates). Three of the one-to-one crosses established mate-pair infections. Only one of the five-to-five crosses successfully established, yielding a single mate pair in one animal. Eighteen one-to-one crosses were attempted in the second experiment, with six mate pairs successfully establishing. Cumulatively, nine out of twenty-eight one-to-one crosses were established successfully (32% success rate). Only a single five-to-five cross established a mate pair (11% success rate), suggesting a theoretical maximum number of parasites that can be encapsulated and transplanted successfully.&nbsp;

The ability to generate crosses of populations with different phenotypes is essential to map and identify genes that underlie a phenotype of interest. Here, we demonstrated that gelatin capsules are an inexpensive, minimally invasive method for establishing genetic crosses of ascarids in the chicken host. Despite a lower than 100% success rate, chickens enable many crosses to be attempted at lower cost and on a larger scale than in other host-parasite systems, such as *Haemonchus contortus* crosses in sheep. Future studies should focus on methods to improve success rates, minimizing the number of host animals needed per successful cross and increasing the level of experimental replication. Overall, we demonstrated that genetic crosses can be performed in a model ascarid system using cost-effective methods, enabling the exploration of the genetics underlying traits such as establishment differences, drug efficacy, inheritance, and gene interactions between genetically and phenotypically distinct populations. Development of an ascarid model in a poultry host is ongoing. However, the ability to perform genetic crosses is an important first step towards ascarid genetics.&nbsp;

## Methods

***Poultry***

28 and 36 one-day-old M99 X Ross 308 chicks were received from Longenecker's Hatchery (Elizabethtown, PA) for experiments one and two, respectively. Animals were allowed to acclimate for one week before infection. A starter/grower ration and water were administered *ad libitum* . All animal research was completed under an approved IACUC protocol (AV23A326).

***Parasite infections***

After one week of acclimation, eight donor animals for the first experiment and 16 donor animals for the second experiment were infected with *Ascaridia galli* embryos collected from a previous study (Collins et al., 2025). Each animal was infected with approximately 100 infective-stage embryos delivered into the crop by oral gavage (Collins et al., 2019).

***Necropsy***

Three weeks post-infection, donor animals were humanely euthanized using carbon dioxide asphyxiation, followed by cervical dislocation. The small intestine was then opened laterally, and all contents were scraped into an individual petri dish. Fourth-stage larval parasites were carefully collected from intestinal contents, separated by sex, and the total number of parasites was recorded. Recovered parasites were placed in 0.85% saline and incubated at 37°C for no more than one hour until gelatin capsules were created.

***Crossing***

To perform each cross, one male and one female of the same isolate, or five males and five females of the same isolate, were transferred to the bottom half of a size 5 gelatin-based capsule (XPRS Nutra, South Jordan, Utah). 40 μL of physiological saline was added to the capsule, and the top half was replaced on the capsule. Additional crosses for the same isolate were prepared in the same way, with as many crosses attempted as possible. Prepared capsules were then immediately delivered to the back of the oral cavity of a recipient animal, and the animal was confirmed to have swallowed the capsule. Confirmation of cross success was performed three weeks later using necropsy and recovery of parasites, as detailed above. Females from successful crosses were then dissected for embryos.

***Embryo recovery***

Using dissection scissors, the head of a female ascarid recovered from a successful cross was removed. Holding the tail with tweezers, the ovaries were removed by using a blunt probe to push the internal organs out of the opening at the head. Ovaries filled with embryos were then transferred to a clean petri dish and opened to recover the embryos. Embryos were stored in a 40 mL breathable culture flask in a total volume of 10 mL and maintained for future experiments.

## Data Availability

Description: Worm and cross recovery data from each infected animal.. Resource Type: Dataset. DOI: https://doi.org/10.22002/n50f1-qm642
